# Comparative occurrence pattern of fractures in cattle and buffaloes

**DOI:** 10.14202/vetworld.2019.1154-1159

**Published:** 2019-07-28

**Authors:** Ganga Prasad Yadav, Vandana Sangwan, Ashwani Kumar

**Affiliations:** Department of Veterinary Surgery and Radiology, College of Veterinary Science, Guru Angad Dev Veterinary and Animal Sciences University, Ludhiana, Punjab, India

**Keywords:** buffalo, cattle, fracture, incidence, long bone

## Abstract

**Background and Aim:**

Dairy animals play an important role in the Indian economy. Knowledge of the occurrence pattern of long bone fractures in bovine may help in strategizing the possibilities of treatment and prognosis. This study aimed to find out the comparative occurrence pattern of fractures in cattle and buffaloes.

**Materials and Methods:**

A total of 278 fractures of bovine (171 in cattle and 107 in buffaloes), presented to the Department of Veterinary Surgery and Radiology, Guru Angad Dev Veterinary and Animal Sciences University, Ludhiana, Punjab, India, during a study of 1 year, were investigated for the occurrence pattern, in relation to species, age, body weight, gender, bone involved, type of fracture (closed or open), and the presentation of bovine as standing or in recumbent state.

**Results:**

The overall hospital occurrence of fractures in bovine was 4.24% and most of the fractures resulted from slipping and falling on hard floor. Of 278 fractures, the majority (90.28%) involved long bones (n=251; 103 buffaloes and 148 cattle). Forelimb fractures were recorded more common in buffaloes (64.08%), whereas the cattle suffered more of hind limb fractures (60.23%). Cattle also included 11 cases of bilateral fractures of metacarpal (n=3), tibia (n=1), radius and ulna (n=1), and mandible (n=6). Fracture of olecranon constituted 6.83% (n=19) and majority (n=15) were in buffaloes. The cattle had higher percent of open fractures (54.38%) as compared to that in buffaloes (17.76%). Marginally higher percentage of cattle (33.33%) suffering from fracture were recumbent as compared to buffaloes (23.36%) and femur fractures were found to be a leading cause of recumbency (100% in cattle and 75.00% in buffaloes).

**Conclusion:**

Species-specific differences in the occurrence pattern of fractures exist among cattle and buffaloes. Cattle are found to be more susceptible (1.5 times) to fractures as compared to buffaloes. The buffaloes are vulnerable to forelimb fractures while the cattle to the hind limb. As compared to forelimb, long bone fractures of hind limb are more commonly associated with recumbency in bovine. Cattle are more prone to mandible fractures and the open fractures of long bones as compared to buffaloes.

## Introduction

Cows and buffaloes are major milk-producing animals. Fractures are commonly encountered in bovines, resulting from a self-inflicted trauma or external factors such as herd mate or farm machinery [1]. The prognosis depends on the severity of injury (open or closed), bone involved, location, and type of fracture [2].

Data on the occurrence of fractures in cattle are available [2,3], but there is lack of literature on the comparative occurrence pattern of fractures in cattle and buffaloes. Although cows and buffaloes appear similar, morphologically, these are two different species. Both differ from each other in many aspects such as behavior, physiology, susceptibility to various disease conditions, and difference in the clinical manifestations [4]. Therefore, it was hypothesized that the occurrence and pattern of fractures in cattle and buffaloes may vary that could have potential implications in strategizing the possibilities of treatment and prognosis.

The objective of this study was to find out the comparative, referral hospital based, occurrence pattern of fractures in cattle and buffaloes.

## Materials and Methods

### Ethical approval

This study was conducted at a Referral Veterinary Teaching Hospital of College of Veterinary Science for a period of 1 year (January 2017-December 2017) and was duly approved by the Institutional Animal Ethics Committee of Guru Angad Dev Veterinary and Animal Sciences University, Pujab, India.

### Study animals

All the bovines presented to the referral veterinary teaching hospital with radiographically, confirmed fractures were included in this study. The study excluded fractures of pelvis, scapula, or the spine as they could not be confirmed, radiographically. The age, body weight, gender, bone involved, type of fracture (closed or open), and the presentation of animal as standing or in recumbent state were recorded in all the bovines. The month/season-wise occurrence and the pregnancy status of the ailing bovines were also recorded.

### Statistical analysis

The quantitative data generated were subjected to statistical analysis using Microsoft Excel 2010. The mean and the standard deviation of all the numerical parameters were calculated in all the bovines. The subjective data were analyzed using percentage basis; relative or absolute value.

## Results

Of a total of 7399 cases of farm animals registered at the Referral Veterinary Teaching Hospital during 1 year study period, 88.68% (n=6562) constituted bovine that included cattle (n=3514; 53.55%) and buffaloes (n=3048; 46.45%). The hospital population of cattle, during the period of the study, was 13.26% higher than that of the buffaloes.

Out of 6562, a total of 278 cases of fractures were recorded in bovine (cattle 61.51%, n=171 and buffaloes 38.49%; n=107) which constituted overall occurrence of 4.24%. The percent of fractures in cattle was 37.43% (171-107/171) more than that of in buffaloes. Besides, cattle also included 11 cases of bilateral fractures of metacarpal (n=3), tibia (n=1), radius-ulna (n=1), and mandible (n=6). Interestingly, none of the bilateral fractures were encountered in buffaloes during the period of study. Majority of the bovines presented with fractures were females (83.13% cows and 92% buffaloes) (Tables-1 and 2).

**Table 1:** The occurrence of fractures in buffaloes in 1 year (Mean±Standard deviation).

Buffaloes	Metacarpal	Metatarsal	Femur	Tibia	Radius-ulna	Humerus	Phalanx	Tarsal	Olecranon	Mandible	Total
Number of cases presented	27	10	12	15	8	16	2	2	15	0	107
Age in years (range)	5.14±2.34 (0.5-9)	2.41±1.73 (0.5-6)	2.38±1.7 (0.67-6)	2.73±2.71 (0.083-8)	2.81±2.49 (0.67-7)	3.72±1.7 (1.5-8)	6±2.82 (4-8)	6.75±7.42 (1.5-12)	4.47±1.29 (2.5-7)	0	
B. wt. in kg (range)	399.26±144.59 (85-550)	271.5±156.49 (70-500)	240.00±145.97 (50-470)	229.67±168.48 (65-500)	281.87±147.55 (150-470)	370±109.85 (100-500)	413.5±19.09 (400-427)	300±141.42 (200-400)	408±62.93 (250-550)	0	
Gender F: M	25F: 2M	9F: 1M	11F: 1M	13F: 2M	7F: 1M	15F: 1M	2F	2F	15F	0	99F: 8M
Simple ^#^’s	20	5	12	12	5	16	2	1	15	0	88
Compound ^#^’s	7	5	0	3	3	0	0	1	0		19
Recumbent	0	6	9	4	1	3	0	2	0	0	25
Standing	27	4	3	11	7	13	2	0	15	0	92

B. wt.: Body weight

**Table 2:** The occurrence of fractures in cattle in 1 year (Mean±Standard deviation).

Cattle	Metacarpal	Metatarsal	Femur	Tibia	Radius-ulna	Humerus	Phalanx	Tarsal	Olecranon	Mandible	Total
Number of cases presented (+bilateral)	18+3	47	10	45+1	13+1	6	6	2	4	9+6	160 cases 171 bones
Age in years (range)	4.35±5.13 (0.25-7)	3.25±2.34 (0.01-8)	2.65±1.58 (1-5)	3.31±3.22 (0.05-10)	4.61±3.27 (0.0082-12)	1.77±2.71 (0.041-7)	3.67±1.75 (1-6)	3.75±3.18 (1.5-6)	41.15±1.15 (3-5)	3.25±2.74 (0.0055-7)	
B. wt. in kg (range)	316.33±164.77 (60-600)	296.13±138.33 (27-500)	264.5±153.7 (40-400)	277.00±154.16 (35-500)	341.15±139.37 (40-500)	168.33±179.6 (40-400)	340±112.26 (120-400)	275.0±247.48 (100-450)	400±16.33 (380-420)	267.22±167.58 (35-400)	
Gender F: M	13F: 5M	44F: 3M	9F: 1M	35F: 10M	11F: 2M	6F	3F: 3M	2F	3F: 1M	7F: 2M	133F: 27M
Simple ^#^’s	10	10	10	20	9	6	6	0	4	3	78
Compound ^#^’s	11	37	0	26	5	0	0	2	0	12	93
Recumbent	5	15	10	20	4	2	0	1	0	0	57
Standing	13	32	0	25	9	4	6	1	4	9	103

B. wt.: Body weight

In buffaloes, metacarpals were the most fractured bone (n=27, 25.23%) followed by humerus, tibia, and olecranon (Table-1), whereas, in cattle, the metatarsals (n=47; 27.48%) and tibia (n=46; 26.90%) were the most affected bones, followed by metacarpals, mandible, and radius-ulna (Table-2).

No mandible fracture was recorded in the buffaloes while a total of nine cases of mandible fractures were recorded in cattle, of which six were bilateral. The reasons for mandible fractures in cattle were traction during delivery and vehicular accident.

Another important difference observed in the pattern of fractures among cattle and buffaloes (Tables-1 and 2) was that forelimb fractures were more common in the buffaloes (64.08%), while the cattle had more percent of hind limb fractures (60.23%). Most of the fractures encountered were due to slipping and falling on the hard floor.

The overall average age of cattle and buffaloes suffering from fractures was 3.47 years and 4.05 years, respectively, with the minimum average age 1.77 years observed in the humerus fractures of cattle and 2.38 years in the femur fracture of buffaloes. Bovines of all age ranges were presented with all type of fractures except the olecranon fractures, where all the affected bovines were adult or sub-adult (3-5 years). Besides, the olecranon fractures were presented more in buffaloes (15/19=78.94%) as compared to cattle (Tables-1 and 2).

In cattle, almost equal number of fractures was open (54.38%) and closed (45.61%), whereas in buffaloes, the majority of the fractures were closed (82.24%). In this study, 23.36% (n=25) of buffaloes and 33.33% of cattle (n=57) with fractures were presented in recumbent state (Tables-1 and 2). Fracture of the femur was found to be associated with recumbency in 75.0% (9/12) of buffaloes and 100% of cattle (10/10).

While comparing the month/season-wise occurrence pattern of fractures, it was observed that the maximum number of fractures in buffaloes was recorded in April and May months, followed by July, January, and February and the least in August (Table-3). However, in cattle, there was no specific pattern of distribution, only that the minimum number of fractures (n=8) was presented in October. The remaining months had almost equal distribution of fracture cases (11-17/month). When comparing season-wise occurrence, cattle had almost equal distribution; the winter season (November, December, January, and February) had 55 cases, while summer (March, April, May, and June) had 54 and rainy 51 (Table-4). However, in buffaloes, the summer season had maximum percent of fracture cases (47.66%) followed by the winter (28.97%) and the least in the rainy season (23.36%).

**Table 3:** The month-wise hospital occurrence of fractures in buffaloes.

Month	Metacarpal	Metatarsal	Femur	Tibia	Radius-ulna	Humerus	Phalanx	Tarsal	Olecranon	Mandible	Total
January	1	1	1	1	0	0	0	0	1	0	5
February	2	0	0	0	1	1	0	0	1	0	5
March	1	0	1	2	1	0	1	1	2	0	9
April	4	1	0	1	0	4	0	0	4	0	14
May	2	1	5	1	1	1	0	0	4	0	15
June	2	2	1	3	3	2	0	0	0	0	13
July	0	0	1	0	0	3	1	0	0	0	5
August	1	0	1	0	0	0	0	0	0	0	2
September	2	1	1	0	1	2	0	1	0	0	8
October	0	2	1	1	1	3	0	0	2	0	10
November	8	1	0	2	0	0	0	0	0	0	11
December	4	1	0	4	0	0	0	0	1	0	10
Grand total	27	10	12	15	8	16	2	2	15	0	107

**Table 4:** The month-wise hospital occurrence of fractures in cattle.

Month	Metacarpal	Metatarsal	Femur	Tibia	Radius-ulna	Humerus	Phalanx	Tarsal	Olecranon	Mandible	Total
January	1	2	1	4	2	0	0	0	0	1	11
February	4	5	2	2	0	0	1	0	2	1	17
March	0	3	1	8	0	1	0	0	0	1	14
April	2	6	1	1	1	2	1	0	1	0	15
May	0	2	2	5	1	0	1	0	0	1	12
June	1	5	0	4	0	0	2	0	1	0	13
July	2	3	1	6	0	1	0	0	0	0	13
August	2	4	1	4	1	0	0	0	0	3	15
September	2	8	0	2	1	0	0	1	0	1	15
October	0	2	0	3	1	0	0	1	0	1	8
November	3	3	1	4	2	2	0	0	0	0	15
December	1	4	0	2	4	0	1	0	0	0	12
Grand total	18	47	10	45	11	6	6	2	4	9	160

In this study, only 12 bovines (4.32%) were in advanced stage of pregnancy with 10 buffaloes (4 Metacarpal+1 Tibia+3 Humerus+2 Olecranon) and two cows (1 Metatarsal+1 Tibia) (Tables-1 and 2), suggesting that advance stage of pregnancy may not be an important predisposing factor for long bone fractures in the bovines.

## Discussion

The hospital occurrence of fractures in bovine has been reported to vary from 6.06 to 9.66% by various workers [5,6] which were relatively higher as observed in the current study. All the bilateral fractures, in the present investigation, were observed in the cattle only and none in buffaloes. However, a few sporadic case studies of bilateral/ipsilateral fractures in buffaloes have been reported in literature [7].

In this study, the occurrence of metatarsal fractures was observed to be almost double the frequency than the metacarpal in the cattle which is contrary to the earlier findings [3,8,9] who stated that the metacarpal fractures occur in about double the frequency than the metatarsal fractures in cattle. One reason for this might be that the high percent of metacarpal fractures reported is in the neonatal calves, due to traction during assisted delivery [9,10], but in the current study, only two cases of trauma due to traction were recorded (one metacarpal and one metatarsal).

The cattle and the buffaloes represented different number and type of bone fractures, which might be due to the difference in anatomy and physiology, management practices, behavior, or production status between the cattle and buffaloes. The percent of tibia fractures in the cattle of this study was 27.48%, while Tulleners [3] reported the incidence to be 13.3%. The metacarpal and the metatarsal fractures count has been reported to be approximately 50% of all the fractures in cattle [8,11], while in the present study, it was 37.05%. The highest numbers of metacarpal and metatarsal fractures in cattle are reported to be followed by femur and tibia a fracture [12] which was not true for this study. However, Gangl *et al*. [5] reported 50% fractures to be of tibia in cattle and metatarsal and metacarpal to be less.

A species-wise difference in the percent of open/compound fractures was recorded in the present study, with the cattle having more of open fractures in comparison to that of buffaloes. The reason for this difference might be due to the thin skin and tender muscles of cattle, which break down easily, compared to buffaloes. Compound/open fractures have guarded prognosis in cattle [3]. Poor prognostic bovines may require limb amputation or euthanasia [2]. Adequate and timely first aid to the injured bovine with distal limb fracture may prevent it from becoming open and thus have better prognosis.

A species-wise difference in the occurrence pattern of fractures in the bones of fore and hind limb was recorded in the present study, with the buffaloes having more of forelimb and cattle having more of hind limb bone fracture. The majority of the olecranon fractures were reported in buffaloes. This could be attributed to the difference in the distribution of their body weight or weak hind quarter of cattle of this region.

The gender-wise variation in the occurrence of fractures in cattle and buffaloes was recorded in this study, with the males of cattle to be presented more than that of the buffaloes. It may be due to the reason that most of the male cattle in the region of study are left on road for livelihood. Such cases may suffer from fractures due to road accidents. However, cited literature report male cattle population to have more metacarpal fractures due to traction during dystocia [9,10,13].

Gangl *et al*. [5] reported the maximum occurrence of fractures in cattle to be in the months of April, May, and June (35.35%) and the reason stated was that it is the season after calving when calves go out in the pastures for the 1^st^ time and undergo injuries, but in this study, the buffalo calf population was low and also no pasture feeding practice is followed in the region of study. Moreover, in the current study, the presentation of maximum number of fractures in the April and May months (summer season) in buffaloes was not related to calving.

## Conclusion

- Cattle are more susceptible (1.5 times) to fractures as compared to buffaloes
- Buffaloes are more prone to forelimb fractures, while cattle to the hind limb
- As compared to forelimb, long bone fractures of hind limb predisposed bovine to recumbency
- Cattle are more prone to mandible fractures in comparison to buffaloes
- Cattle are more prone to open fractures of long bones in comparison to buffaloes.

## Authors’ Contributions

The research was conceived by VS. The data collection and compilation were done by GPY. The radiographic interpretation was done by VS and AK. The manuscript was written by VS and revised by AK. All the authors have read and approved the final manuscript.

## References

[1] Velavan A, Sivaraman S, Krishnakumar K (2014). Successful management of a compound fracture in a buffalo using a fabricated polyvinylchloride splint in a field setting. Buffalo Bull.

[2] Jean G.S, Anderson D.E (2014). Decision analysis for fracture management in cattle. Vet. Clin. North Am. Food Anim. Pract.

[3] Tulleners E.P (1986). Management of bovine orthopedic problems, Part I:Fractures. Compend. Contin. Educ. Pract. Vet.

[4] Sangwan V, Mohindroo J, Kumar A, Randhawa C.S (2018). Clinical, radiographic and ultrasonographic differences in the cows and buffaloes suffering from pericarditis. Int. J. Livest. Res.

[5] Gangl M, Grulke S, Serteyn D, Touati K (2006). Retrospective study of 99 cases of bone fractures in cattle treated by external coaptation or confinement. Vet. Rec.

[6] Singh D, Singh R, Chandrapuria V.P, Vaish R (2017). Occurrence pattern of different types of fracture in bovine, caprine and canine. J. Anim. Res.

[7] Prabhakar V, Raghunath M, Singh S.S, Mohindroo J, Singh T, Verma P (2012). Clinical management of metacarpal and metatarsal fractures in two buffalo calves. Intas Polivet.

[8] Steiner A, Iselin U, Auer J.A, Lischer C.J (1993). Physeal fractures of the metacarpus and metatarsus in cattle. Vet. Comp. Orthop. Traumatol.

[9] Arican M, Erol H, Esin E, Parlak K (2014). Retrospective study of fractures in neonatal calves:181 Cases (2002-2012). Pak. Vet. J.

[10] Akin I (2017). Calf metacarpal fractures in association with bovine dystocia:Case series among calves. Ataturk Univ. J. Vet. Sci.

[11] Anderson D.E, Jean G.S (2008). Management of fractures in field settings. Vet. Clin. North Am. Food Anim. Pract.

[12] Crawford W.H, Fretz P.B (1985). Long bone fractures in large animal a retrospective study. Vet. Surg.

[13] Belge A, Akin I, Gülaydin A, Yazici M.F (2016). The treatment of distal metacarpus fracture with locking compression plate in calves. Turk. J. Vet. Anim. Sci.

